# Effectiveness of an online training course to improve evidence-based leadership practices among unit leaders: study protocol for a cluster randomised two-arm controlled trial (EVILEAD)

**DOI:** 10.1186/s13063-025-09207-9

**Published:** 2025-11-14

**Authors:** Maritta Välimäki, Maija Satamo, Min Yang

**Affiliations:** 1 Department of Public Health, University of Helsinki, PL 20, Tukholmankatu 8 b, Helsinki, 00014 Finland; 2 Helsinki University Hospital, Helsinki, Finland; 3 Faculty of Medicine, Department of Nursing Science, University of Turku, Turku, 20014 Finland; 4 Faculty of Health, Art and Design, Swinburne University of Technology, Victoria, 3122 Australia

**Keywords:** Evidence-based leadership, Cluster randomised controlled trial, Leaders, Training

## Abstract

**Background:**

A substantial knowledge base exists on how nurse leaders can support clinical staff in implementing evidence-based knowledge into practice. However, fewer studies are available on how leaders themselves learn and use evidence in their daily work. This study aims to evaluate the effectiveness of an online training course designed to improve the skills of unit leaders by enhancing individual outcomes through evidence-based practice (EBP), team-based outcomes through EBP implementation, and organisational outcomes through the quality of care.

**Methods:**

The effectiveness of the online training course will be evaluated using a cluster randomised two-arm controlled trial. Participants will be unit leaders recruited from hospitals in the Helsinki and Uusimaa Hospital District in Finland. At least 12 units will be represented, with approximately 25 participants in each unit (6 units in each arm). The sample for analysis will consist of at least 300 staff members. In the intervention arm, participants will be invited to join the online leadership course, while participants in the control group will continue practice as usual. The primary outcome is evidence-based practice, while the secondary outcomes are self-esteem, self-efficacy, implementation of EBP, quality of care, as well as work absence and intention to leave. The effectiveness of the intervention on the primary and secondary outcomes will be assessed based on the intention-to-treat principle. Feasibility data will be analysed separately. Sample size calculations are based on preliminary power analyses.

**Discussion:**

To the best of our knowledge, this is the first study to evaluate the effectiveness of an online course on evidence-based knowledge using a full cluster randomised study. By applying evidence-based practices, unit leaders can ensure that clinical decisions are grounded in the latest study results, which will lead to more effective decision-making.

Trial registration

ClinicalTrials.gov, NCT06886581. Registered on 14 March 2025, https://clinicaltrials.gov/study/NCT06886581

**Supplementary Information:**

The online version contains supplementary material available at 10.1186/s13063-025-09207-9.

## Background

The adoption, implementation, and sustainment of evidenced-based practices (EBPs) are increasingly important for health and allied healthcare organisations globally [1]. In Finland, the effectiveness and quality of healthcare and patient safety are key goals of the Ministry of Social Affairs and Health [2]. Managerial-level healthcare workers play an important role in supporting the implementation of evidence-based knowledge into clinical practice [3] by influencing the knowledge, behaviours, decisions, and communication among staff [4]. At the same time, as leaders, managers may hinder the implementation of EBP if their own knowledge or previous experience of working with EBP principles is limited or if they hold negative attitudes towards EBP [5].

There is a growing trend toward organising online leadership training; however, assessments of their effectiveness and cost-effectiveness remain in their infancy, primarily employing quasi-experimental study designs. For instance, Clark et al. [6] evaluated the acceptability and preliminary outcomes of a scalable online learning program aimed at enhancing the leadership competencies of midwives (Leadership Link). While participant satisfaction was reported to be high, only 42% (*N* = 186) completed the full curriculum, and only 76 (40.9%) provided both pre- and immediate post-program survey data. Similarly, Ortega et al. [7] assessed learner performance in an online leadership course involving 289 individuals from 41 countries. Despite receiving more enrolment applications than any other course on the virtual campus, only two-thirds (63.5%) completed the course, and 48.7% rated it. Non-completers cited being too busy with other obligations, encountering technical issues, or finding the course content irrelevant or excessively lengthy. Furthermore, Luo et al. [8] investigated the impact of assigned leadership and its key characteristics on promoting team learning in an online context using a quasi-experimental design with 94 university students. The study found no significant behavioural increase among assigned leaders in reading and reflection tasks, suggesting a moderating effect of learning tasks on leadership impact.

Although there is a substantial knowledge base on how leaders can support staff in implementing evidence-based knowledge into clinical practice, there are far fewer effectiveness studies on how leaders themselves learn and use evidence in their daily work [9]. Therefore, more studies with rigorous research designs are needed to capture the complexity of interventions in improving leadership abilities [10].

This cluster randomisation study will evaluate the effectiveness of an online training course, designed to target unit leaders. The intervention will be delivered at the cluster level, i.e. professionals within clusters (education of unit leaders) [11]. The cluster randomisation study design fits our intention to enhance the application of evidence within entire unit teams, including unit leaders and staff members [12]. Using cluster randomisation will help prevent contamination, which could occur if study participants within the same unit were to be randomised to different groups [13].

### Objectives and hypotheses

The overall aim of the study is to evaluate the effectiveness of an online training course, designed to target unit leaders, to improve EBP skills and implementation as well as the quality of care.

The objectives are as follows:

#### Primary objective

1. To evaluate the effectiveness of the online training course on staff’s evidence-based practice.

#### Secondary objectives

2. To evaluate the effectiveness of the online training course on staff’s self-esteem and self-efficacy.

3. To evaluate the effectiveness of the online training course on leadership for EBP implementation at the organisational level.

4. To evaluate the effectiveness of the online training course on the quality of patient care.

5. To evaluate the effectiveness of the online training course in reducing staff’s absence (sick leave, intention to leave the unit or hospital).

##### ***Hypotheses***

Primary hypothesis:

1. In units where leaders attend the evidence-based online course, staff will have higher scores in evidence-based practice after the course than in the units where leaders do not attend the course.

Secondary hypotheses:

2. In units where leaders attend the evidence-based online course, staff will have higher self-esteem and self-efficacy after the course than in the units where leaders do not attend the course.

3. In units where leaders attend the evidence-based online course, staff will have better EBP implementation scores after the course than in the units where leaders do not attend the course.

4. In units where leaders attend the evidence-based online course, the quality of patient care will be higher after the course than in the units where leaders do not attend the course.

5. In units where leaders attend the evidence-based online course, staff’s work absence and intention to leave the unit or profession will be lower than in those units where leaders do not attend the course.

#### Process evaluation objectives

Feasibility and fidelity of the intervention will be assessed as well as the number of times participants log onto the learning platform, the overall number of completed course tasks, the number of tasks completed by each participant, and the drop-out rate by the number of participants who leave the study early (dropped-out from intervention, no follow-up data). Despite the importance of assessing acceptability and adherence when evaluating an assigned intervention, these qualities are rarely measured and reported in cluster trial publications [13].

## Methods

### Trial design

This study will follow a pragmatic, parallel group (1:1), two-arm cluster randomised, superiority trial design, which aims to compare the effectiveness of an online training course, targeting unit leaders, to improve EBP skills and implementation among staff as well as the quality of care (2025–2028). The Consolidated Standards of Reporting Trials (CONSORT 2010) statement extension to cluster randomised trials was used in designing the study [14]. The SPIRIT checklist [15] guided the study methodology (Additional File 1), and the GREET guideline was consulted when planning this educational EBP intervention [16]. To support the open approach of the trial, the study has been registered at ClinicalTrials.gov (NCT06886581).

To ensure the feasibility of the study design, the study protocol has already been tested in other studies in two countries, Finland and China [17, 18]. The data from these feasibility studies will not be used in this new study.

### Setting

In Finland, the responsibility for organising healthcare services lies with the 21 wellbeing services counties. These counties coordinate the delivery of health and social care services to ensure comprehensive and equitable access for residents within their respective regions [19]. This cluster trial will be conducted in the region of Uusimaa. In 2022, the health and social services sector experienced a higher rate of job growth than other sectors, making it the largest employing industry in Finland [20]. According to national health care statistics in 2023, Finland employed 5138 unit leaders and 77,105 nurses (including registered nurses, midwives, public health nurses, and paramedics with a bachelor’s degree) [21].

#### Eligibility of the clusters and individual participants

For a cluster to be eligible for participation, it should comprise a unit providing specialised or primary care with at least 20 staff members (unit leaders and other staff) and be interested in joining the study. A ‘unit’ here refers to a ward, department, or any independent operational entity that has its own immediate leader or supervisor. Units will be excluded if they have low staff capacity (fewer than 20 total staff members), if they do not provide treatment to patients (e.g., supply management, laboratory), or if they are expected to undergo significant operational changes or closures.

The online training course is designed to target unit leaders working in the social or health care sector in a managerial position. Unit leaders were selected as the primary target group of the course because they represent important first-level actors in the implementation process of evidence-based knowledge into clinical practice [22]. To be eligible for participation, unit leaders should 1) have a professional licence to work in the health or social care sector, 2) have an official managerial position at the study organisation, 3) demonstrate a willingness to join the study, 4) represent any gender, 5) be able to speak, read, and write in Finnish, 6) and give (individual level) informed consent to join the study. Unit leaders with a background in nursing or medicine (doctor, dentist) should hold positions in strategic, middle, and supervisory management within social and healthcare units [23]. Unit leaders on long-term sick leave or study leave will be excluded.

In Finland, there are no legal regulations for management and supervisory positions; these are defined by the employer [24]. However, in social and health care positions, the minimum requirement is a bachelor’s degree in health care from a university of applied sciences (e.g., registered nurse, physiotherapist, radiographer) and being licensed by the National Supervisory Authority for Welfare and Health [25]. Medical and dental specialists receive training to manage demanding diagnostic methods and to perform in roles of healthcare leadership, administration, planning, interdisciplinary collaboration, guidance of learning, and assessment of competence within their work community [26]. For nurses in Finland, the Union for Health and Social Care Professionals recommends an advanced university degree or a relevant advanced degree from a university of applied sciences for strategic and middle management positions (e.g., administrative chief nurse, chief nurse, or nursing director) [24]. The closer the leader’s role is to patient care, the more critical their nursing expertise and the application of evidence-based practices are [23].

Any staff members (e.g., registered nurses, assistant nurses, or any professional staff members with social and health care degrees) working in the same units as the unit leaders who are willing to join the study will be eligible for participation. Staff members who are off duty (family leave, long-term sick leave, study leave, or any other reason) will be excluded from the study.

To secure the privacy of the unit leaders of the organisations, the Chief Executive Officers in Nursing will act as a contact person for each study organisation. The contact person will screen potential units to determine whether their leaders fit into the following eligibility criteria.

### Interventions

The units will be randomised into two groups: an evidence-based leadership training (experimental) group and a passive control group.

For the evidence-based leadership training (experimental) group, the intervention will be targeted at the cluster level (i.e. tasks included in the intervention will be done in close collaboration with members of the same unit). The participants in the experimental group will join a seven-month online training course aiming to improve evidence-based leadership practice. The structure of the course will follow the steps of the evidence-based approach: [1] each participant will identify their specific unit problem to be solved together with the team and the unit leader will address this problem during the course; [2] organisational data will be collated and analysed to understand the key problem of the unit; [3] scientific literature will be searched for and critically appraised; [4] the views of stakeholders (patients, family members, etc.) will be considered along with implications; and [5] all sources of information will be collated together by the unit leader together with the team, after which the solution will be implemented into practice [17]; the actual situation will be evaluated [27]. Each module includes specific learning material (slideshow presentations, scientific articles), peer-group discussions, clinical exercises, and self-assessment and self-reflection [17]. All course tasks will be arranged so that leaders will work together with their team.

The online training intervention will be run in small groups with unit leaders (about 15 participants in each training group). Tutors (1/group) with an academic and/or healthcare professional background will follow participants’ and their team’s learning process in each small group. Tutors will receive centralised training on the study requirements through a total of 4 h of hands-on training sessions. These sessions will cover topics related to the tutor’s role, which includes carefully monitoring participants’ engagement (returned tasks and course drop-outs, peer discussions, and any other activity), answering practical questions related to the course via group chat and/or email, and giving feedback on all course assignments. Although no makeup sessions will be organised if a participant misses a training session, individual participants (or the tutor) may still request extended time to complete their tasks. However, a unit leader can choose to stop participating in the online course at any time, for any reason, without staff needing to withdraw from the entire study (i.e. the staff can still provide follow-up data). A student may be removed from the online course if, for example, they behave unethically towards other students. At the beginning of the course, the ethical guidelines regarding online education will be presented.

In line with the Finnish National Board on Research Integrity (TENK) guidelines [28], no compensation or reimbursement will be paid to the study participants. No additional support, training, or mentoring related to leadership skills will be offered by the research team after the intervention period.

Details of the assumptions and intervention mechanism, pedagogical approaches, learning process, structure, and content of the course are described in the intervention protocol [17]. In addition, the structure and content of the intervention are described in Additional File 2 using the GREET checklist [16].

No specific activities will be organised for the passive control group participants.

### Outcomes

The data will be formed based on the characteristics of each unit and its participants, outcome data, course feedback, and feasibility data of the online training course.

#### Characteristics of the units and participants

Each unit’s clinical specialty, number of beds, and total number of staff will be collected based on unit records, with the help of the unit leaders and a contact person for each organisation. The researchers will not have access to organisational databases. The age, gender, degrees, position and length of working experience of each participant will be collected as part of baseline data.

#### Outcomes

The outcomes are targeted to evaluate the effectiveness of the evidence-based online course on an individual, team, and organisational level [4]. The primary endpoint with respect to the effectiveness of the training course is improvement in evidence-based practice (EBP) from baseline to follow-up (month 7), measured immediately after the intervention using an electronic survey administered to the unit leaders and staff.

Primary outcome.

*Evidence-based practice*: The Evidence-based Practice Questionnaire (EBPQ) [29] assesses the implementation of evidence-based practice (24 items, score 1–7, three sub-scales), focusing on [1] knowledge/skills (14 items, score 1–7, 1 = Poor–7 = Best, range 14–98, a higher score represents greater knowledge in EBP); [2] attitudes (4 items, score 1–7, range 4–28; a higher score represents a more positive attitude toward EBP); [3] evidence-based practice (6 items, score 1–7, 1 = never, 7 = frequently, range 6–42, a higher score represents more use of EBP). The instrument’s internal consistency has been rated as good, as indicated by a Cronbach’s alpha of 0.87 across the full scale [29].

Secondary outcomes.

The data for the secondary outcomes will be collected at baseline and at month 7.

*Self-esteem*: The Rosenberg Self-Esteem Scale (RSE) [30] evaluates individual self-esteem with a 10-item unidimensional scale by focusing on both positive and negative feelings about the self (4-point Likert scale, 1 = strongly agree–4 = strongly disagree). The sum score ranges 10–40, and a higher score indicates higher self-esteem. The RSE has shown high internal consistency reliability, with an average Cronbach’s alpha of 0.90 [31].

*Self-efficacy*: The General Self-Efficacy Scale (GSE) [32] assesses optimistic self-beliefs in coping with a variety of difficult life demands using 10 items (4-point Likert scale, 1 = not at all true–4 = exactly true). The sum score ranges 10–40, and a higher score indicates better coping abilities. The GSE scale has shown good reliability and unidimensionality across 25 countries, with an overall Cronbach’s alpha of 0.86 [33].

*The implementation of EBP*: The Implementation Leadership Scale (ILS) is available in two versions, one for staff and one for supervisors [22]. The scale comprises a 12-item questionnaire measuring leadership in EBP implementation. It consists of four subscales: 1) proactive leadership, 2) knowledgeable leadership, 3) supportive leadership, and 4) perseverant leadership. It is scored from 0 (not at all) to 4 (to a very great extent), with a higher score representing stronger implementation of EBP. The ILS has demonstrated strong psychometric properties, including face, content, and construct validity as well as internal consistency and convergent validity across multiple health professional groups [34]. Internal consistency reliability has been found to be high, with a Cronbach’s alpha of 0.98 for the total scale and 0.95–0.96 for the subscales [22].

*The quality of care*: Patient death, the number of reported complaints, and medication errors will be collected from hospital records after the training course (to be balanced according to follow-up time).

*Work absence and intention to leave*: The number of days staff are absent during the course (e.g., sick leave) and intention to leave will be measured: [1] “In the last six months, how often have you planned to leave your unit”; [2] “In the last six months, how often have you planned to leave your hospital”; and [3] “In the last six months, how often have you planned to leave your profession” using a five-point scale (0 = never; 1 = now and then; 2 = quite often; 3 = very often; 4 = all the time).

After the course (month 7), course feedback will be collected (8 items developed for the study): [1] “This course was appealing to me”; [2] “I felt happy when I participated in the course”; [3] “Participation in the course required too much effort from me”; [4] “It was worth participating in this course”; [5] “I found this course valuable”; [6] “I was able to meet the requirements of the course”; [7] “This course fits well with my personal values”; and [8] “I would recommend the course to others”. Participants will consider the propositions with five possible answers (1 = completely disagree; 2 = disagree; 3 = neither agree nor disagree; 4 = agree; 5 = completely agree). Possible contamination of the study groups and any perceived adverse event or harm will be screened for during and after the intervention (months 0–7) (yes, no, not sure).

Further, feasibility and fidelity of the intervention will be assessed after course completion (month 7): [1] the number of total logins to each module (*n*); [2] the number of participants who logged in to each module (*n*, %); [3] adherence to the course, measured by the number of returned course tasks out of all possible tasks (*f*, %); [4] adherence to the course tasks, measured by the number of participants who returned course tasks for each module (*n*, %); [5] drop-out from the intervention, measured by the number of participants who leave the intervention early; [6] drop-out from the study, measured by the number of participants who leave the study early.

None of the content of the participants’ learning material, self-reflections, or peer discussion data on the Moodle platform will be analysed in this study.

Important protocol modifications will be communicated to the relevant parties, including the Ethical Review Board and the study organisations. Any modifications will be reported in the trial registry (ClinicalTrials.gov) and in the final report.

**Fig. 1:**
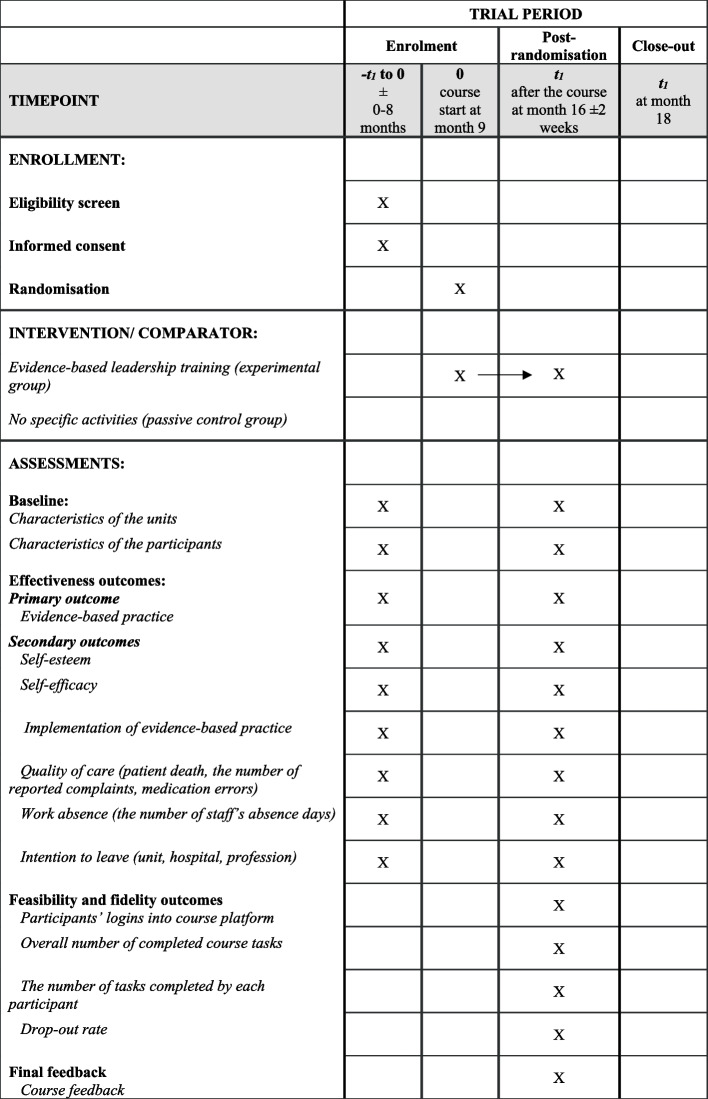
Outcomes, instruments, and specific timelines for outcome assessment using SPIRIT [15]

Outcomes, instruments, and specific timelines for each outcome assessment are described in Fig. 1.

### Sample size

A previous study indicated a 6-point increase in the EBPQ outcome from baseline to a key time point in the intervention group and a 5-point decrease over time in the control group [35]. We assume there will be a 10-point difference in the change of the primary outcome over time between the two groups and an estimated standard deviation of 20 [36]. With a power of 80%, a 5% type I error with a two-sided test, an inter-class correlation (ICC) of 0.03, and an average unit size of 25 with a variation of 1, the estimated sample at the cluster level is 12, 6 in each treatment arm. This would mean a total of 300 participants, 150 in each arm. As in many other trials, we anticipate challenges in recruitment and adherence due to the limited time and resources of nurse staff, trial-specific issues, communication difficulties, inadequate research experience and training, and a lack of incentives [37]. We assume that if 58% of participants complete the post-intervention assessment after the online training [38], the number of staff to be recruited for the study should be 517. Generally, if the average response rate for the baseline is 42% [39], we should aim to approach at least 1,238 nursing staff. The potential number of participants needed for this study is feasible, as the data will be collected in the wide region in south Finland. The power calculation was made using PASS 15 software for a cluster randomised design.

### Recruitment

To minimise risks of recruitment bias, recruitment of the participants will be done before randomising clusters [11]. An invitation to participate in the study will be sent to the supervisors of all units initially interested in joining the research. The email will include a link to study information and electronic informed consent with the Research Electronic Data Capture tool (REDCap) [40]. After a participant gives electronic informed consent, REDCap will lead them to a baseline survey (background information of the participants of the specific units, baseline data). After baseline data collection, unit leaders will be allocated to the study group as their unit.

All staff members who are working in the same unit as the participating unit leaders will be invited to join the study. First, staff members will receive a general invitation email, including a link to a site for baseline data collection, sent by the unit leader (for privacy reasons) to explain the purpose of the study and its practical arrangements. If the staff member is willing to join the study, they will give electronic consent (REDCap) and respond to the electronic surveys; but they do not join online training. Again, after providing baseline data, each staff member will be allocated to the same study group as their unit leader. The invitation email will include an explicit statement that participation is entirely voluntary, that non-participation will not have any consequences for staff, and that all responses will remain confidential. Participants will also be informed about the study’s purpose, procedures, and any potential risks or benefits. It is emphasised that staff members are not compelled to participate against their will or under pressure. Since the link to the data collection site that will be sent by the unit leader is general (non-specified with staff member’s name), the unit leaders will not be aware of who is joining the study or who is not. In addition, the study invitation will be framed as an opportunity for professional development or improvement rather than a requirement, making it less likely to be perceived as coercive.

To enhance recruitment and reach the target sample size, reminders will be sent via email to potential participants. If necessary, recruitment will be expanded to new locations and departments.

**Fig. 2:**
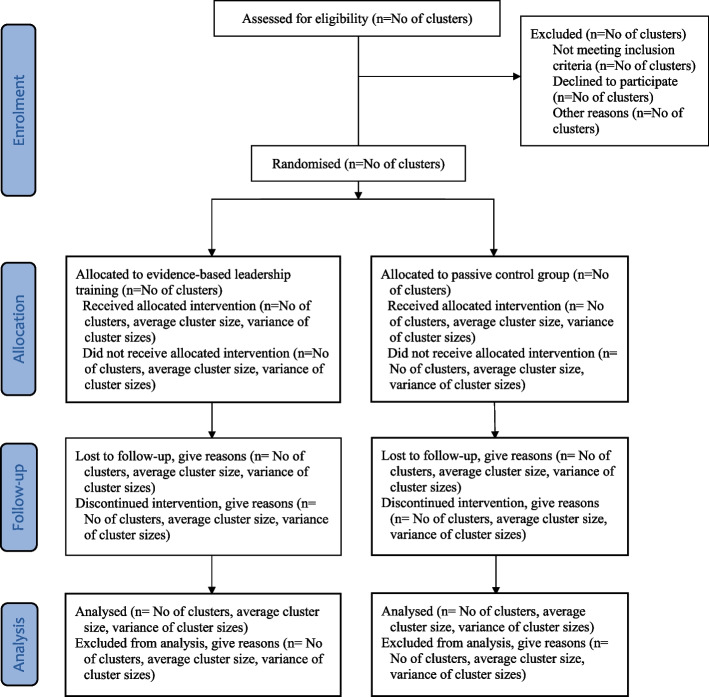
Flow chart [14]

An overview of the trial profile is described in Fig. 2.

### Randomisation

Randomisation in this study will be conducted at the unit level (cluster), not at the hospital level. Units participating in the study will be randomly assigned (1:1) to one of two parallel groups: either the evidence-based leadership training group or the passive control group. This will be done using a computer-generated randomisation list developed by an independent trial statistician. To ensure balance between the study groups, units will be stratified based on the number of staff members in each unit as follows: (I) small units and (II) large units, determined by the median number of staff members in each unit. The randomisation will be generated using SAS software, Version 9.4 of the SAS System for Windows (SAS Institute Inc., Cary, NC, USA).

### Blinding and masking

The investigators who will enrol the units (clusters) will not be able to foresee the assignment. The allocation for each site will be kept blinded, and the staff of each unit will complete the baseline data collection before unit randomisation. When the training coordinator is ready to implement the training for the staff, the statistician will send the allocation groups by email directly to the training coordinator.

Due to the type of intervention, allocation results will be unmasked after randomisation to the unit leaders, contact persons for each unit, staff of the units, the training coordinator, and the researchers participating in the intervention design and implementation; this will reflect a real-world situation.

The leaders whose units are randomised to the intervention group will be registered on the Moodle platform by the training coordinator. Investigators running the preliminary analysis for the Data Management Committee (DMC) will be masked to data until investigators release the database. The statisticians will be masked to unit allocation in each arm.

### Interim analyses and stopping rules

The Data Management Committee (DMC) will be established for the study. The Data Management Committee will be an independent group separate from the sponsor, with no competing interests [41]. The Committee members will represent different disciplines and areas: nurse association, health services, statistics, and ethics, for example. The role of the Committee will be to ensure the quality of the data, data collection, data privacy, safety of the intervention, incorporation of participant feedback, and data analysis and to provide further recommendations for the study [41]. A Trial Steering Committee (TSC) will be established. The role of the TSC will be to oversee the study, make decisions based on the recommendations of the Data Monitoring Committee (DMC), endorse actions, and provide advice on daily operations. Its members will include both independent experts and members of the research team [42].

In addition, day-to-day coordination has already been established to manage the operational aspects necessary to ensure the smooth execution of the research project and to maintain responsibilities that will help keep the study on track and aligned with its objectives. This includes weekly meetings with research team members, as well as planning and scheduling (e.g., recruitment, data collection, data management, and analysis), team communication (e.g., organising meetings, providing updates, ensuring systematic information flow, and ad-hoc communication), recruitment and data management (e.g., data privacy, storage, and management according to protocol), ethics, resource management, and progress monitoring (milestones, delays, achievements), as well as reporting and documentation, staff training, and problem-solving.

The trial conduct will be monitored internally by the research team, with meetings scheduled twice a month to ensure adherence to the protocol and ethical standards. Additionally, the DMC oversees trial conduct, maintaining data integrity throughout the trial period (meeting scheduled twice a year).

Only study investigators, i.e. the Principal Investigator (MV), researcher (MS), and statistician (MY) will have access to the data. No interim analysis will be conducted. The results of the baseline data analysis will be shared and discussed with the DMC. If any significant external factors arise, such as nursing strikes, major organisational changes that alter the unit leaders’ roles, or severe resource shortages within the participating organisations that threaten the study’s implementation or participant recruitment, the trial may be terminated. The final decision to stop the trial will be made by the TSC based on the recommendations of the DMC.

Code breaks will occur only in exceptional circumstances, for example, if during the analysis, the statisticians detect any harm in the data scores. However, this will be done only after discussions with the TSC and based on recommendations of the DCM.

No adverse effects are anticipated due to the educational nature of the intervention. At the end of the intervention, all participants will be systematically asked about any perceived burden or harm caused by the intervention. If participants have perceived (ad-hoc or based on systematic assessment) any unintended effects or burden during or at the end of the intervention, they will be advised to seek care and support from the organisation’s occupational health services. All reports will be reviewed by the principal investigator and discussed during DMC meetings.

The specific criteria for stopping the study are as follows: [1] a significant decline in participant engagement or attendance (e.g. less than 20% course participation) may indicate that the intervention is no longer effective or relevant; [2] consistent negative feedback from participants regarding the content, format, or delivery of the intervention may warrant its cessation; [3] the educational intervention may not be financially sustainable due to unforeseen events (e.g. a catastrophe or staff strike); [4] changes in organisational policies, on-the-job training standards, or regulations may necessitate cessation; and [5] the introduction of a similar compulsory educational initiative or program that supersedes the current intervention may lead to its discontinuation.

We have confirmed that the study organisations are not currently conducting or planning any other leadership courses offered by the employer during the 7-month trial. If similar courses are organised, leaders in the intervention and control groups cannot be restricted from attending other leadership courses. Information about staff members’ participation in any on-the-job training will be collected to identify any potential source of contamination or any factors influencing the effectiveness of the intervention. If the study participant has joined any other leadership course, she/he is able to do so, and it will be reported and considered during the data analysis.

### Data management and statistical analysis

All data will be processed in accordance with the European Union’s (EU) General Data Protection Regulation [43]. The data will not be shared with anyone outside the research team or transferred to another register or to another personal data processor. The quality of the data will be ensured through the use of double data entry. The data will be collected with the participants’ informed consent. Electronic informed consent will be obtained using the REDCap tool. Only the Principal Investigator (MV) and the researcher (MS) will have access to the data related to the obtained informed consent digital documents. The participants will have the right to withdraw from the study, to access their personal data, to demand that their data is rectified or deleted, to restrict the processing of personal data, and to lodge a complaint if they believe a violation of applicable data protection laws has taken place [43].

All study-related information in paper format will be stored securely at the research office. All reports, data collection, processing, and administrative forms will be identified only by a coded ID number to maintain participant confidentiality. All records that contain names or other personal identifiers will be archived separately from the study records identified by the code numbers. All databases will be secured with password-protected codes. Any data for monitoring purposes (data monitoring excel) linking participant ID numbers to other identifying information will be stored in a separate, locked file. Baseline and follow-up data collected from the participants will be stored in a secure cloud storage service provided by the IT services of the University of Turku, with access restricted through password protection. The electronic data collected will be managed with REDCap, a secure, web-based software platform used to conduct research studies [40]. Each research personnel member will receive centralised training on the study requirements through a total of 4 h of hands-on training sessions. These sessions will cover topics such as the use of instruments, obtaining informed consent, data collection procedures, safety issues, voluntariness, and the right to withdraw from the study. All team members are trained to perform quality control and anticipate risks. The Principal Investigator (PI) is responsible for planning and overseeing data management and metadata sharing. The researchers will conduct the data collection, processing, analysis, storage, and organisation. Participant retention and upcoming follow-up data collection will be supported with email reminders. Participant retention and follow-up will be monitored using the Moodle learning platform and REDCap.

Data analysis will be carried out on an intent-to-treat (ITT) basis; every effort will be made to collect outcome data from all participants, regardless of whether they completed the intervention. Assumptions of normality of the residuals will be investigated. The characteristics of the units and respondents (staff members) will be summarised with descriptive statistics for each group. The specific metric will be the change between the baseline score and the final score at 7 months. The method of aggregation or the summary statistics will be based on the type of outcomes. Mean score will be used for score measurement such as the EBPQ, self-esteem, self-efficacy, and EBP scales. The Quality of care and Work absence and intention to leave will be summarised using both count and proportions.

All individuals within the cluster will be included in the trial. Possible duplicate measurements will be identified using participant ID codes, email addresses, or monitoring records.

A descriptive summary of the baseline information for both clusters and individuals will be presented first as tables of summary data. For the primary outcome EBPQ, its change from the baseline to the time immediately after the intervention (key time point) will be analysed with mixed effects linear models (respondents nested within units). In such models, the mean difference in EBPQ scores from baseline to the key point of intervention will be compared between the intervention and passive control groups, with random effects among unit leaders being estimated and characteristics of participants and units being adjusted. The changes in the subscales of the EBPQ will be analysed using multivariate mixed effects models in which simultaneous models for the three subscales are analysed jointly to overcome the inter-subscale correlation that might lead to multi-comparison bias [44]. Multiple imputations for the EBPQ and its subscales will be considered if data is missing at random. The same models will be used for the secondary outcomes, such as scores on the RSE, GSE, EBP and other continuous respondent-level outcomes. For unit-level outcomes at the key point, such as death, number of reported complaints, and number of staff’s sick days, generalised linear models will be used to compare the differences between the two groups with adjustment for unit characteristics.

Sensitivity analyses will be conducted for units in which participation in online training is low to evaluate whether intervention fidelity has any effect on the results of the primary outcome. Results will be expressed using rate ratios, odds ratios, or least-squares mean differences with corresponding 95% CIs, depending on the type of outcome.

As our study will be a cluster randomised controlled trial, its analysis requires special consideration of the correlations within and between the clusters. First, in the sample size calculation, a clustering effect was assumed and accounted for as the ICC coefficient. Second, for the primary analysis, the intracluster (intraclass) correlation coefficient (ICC) will be reported as the proportion of the total variance of the outcome that can be explained by the variation between clusters [14]. For data analysis, the mixed effects model or two-level multilevel models that consider clustering effects will be used. In the model analysis, the variation in the outcomes among clusters will be estimated and tested for significance. Third, the overall efficacy of the outcomes will be estimated after considering the clustering effects.

In addition, covariate analysis will be conducted to control for potential confounding factors in statistical modelling. Participants’ age (novice, senior) and gender (male, female, other), status (unit leader, other staff member) and the engagement of course tasks (number of returned tasks by each participant in the intervention group) will be included in the analysis.

Statistical analyses will be performed with the SAS System for Windows, version 9.4 (SAS Institute Inc) and SPSS, versions 25.0 to 27.0 (IBM Corp). Because no adjustments for multiplicity of testing will be made, only 95% CIs will be reported for the secondary outcomes.

## Discussion

The study will generate new knowledge on the effectiveness of an online training course designed for unit leaders, aiming to improve skills in evidence-based practice (individual outcomes), EBP implementation (team-based outcomes), and the quality of care (organisational outcomes). In 2010, *The Lancet*, reporting on 21st-century health professions [45], highlighted the importance of EBHC knowledge, skills, and attitudes. The report also suggested a shift toward transformative learning in the training of healthcare professionals, where memorisation is replaced with critical reasoning to guide the capacity to search, analyse, assess, and synthesise information for decision-making. Although over a decade has passed, the complex question of whether training supports the ability to base leadership on evidence remains unanswered. This trial has been designed to offer new insight into this timely, ongoing question.

Our intervention is based on existing learning technology. Online learning methods are typically easy to use, engaging, and low in cost. If effective, this educational intervention could be used with many staff groups facing challenges in participating in face-to-face courses. The study results could also be applicable in other settings with similar challenges in organising systematic staff training. Most importantly, this is the first educational trial to analyse the feasibility of online training in different health care settings. The views of the stakeholder representatives in previous feasibility studies have been carefully considered as they have added important value to our study. Stakeholder representatives will also serve on the research team to support the accuracy of the study.

## Trial status

The study was first submitted on 14 March 2025 (ClinicalTrials.gov NCT06886581). Recruitment started on 5 June 2025. Recruitment will be completed by 31 October 2025. This is version 2.0 of the full study protocol, dated 22 September 2025.

## Ethics and dissemination

The University of Helsinki Research Ethics Committee in the Humanities and Social and Behavioural Sciences has reviewed the EVILEAD study protocol and given a positive statement on the research plan (reference code 1/2025). Permission to conduct the study will be obtained from the study organisations or wellbeing services counties. The general principles of the Declaration of Helsinki [46] and local ethical regulations will guide the practical arrangements of the study. The Finnish National Board on Research Integrity will offer more detailed guidance to promote the responsible conduct of research and to prevent research misconduct (47). Safe data management will ensure secure data storage. The data will be processed in a fair manner and under the rules of the European Union’s (EU) General Data Protection Regulation [43].

Substantive contributions of each author to the design, conduct, interpretation, and reporting of the clinical trial have been recognised through the granting of authorship of the study protocol. All authors of the protocol manuscript have read and agreed to its content and are accountable for all aspects of the accuracy and integrity of the manuscript, in accordance with the International Committee of Medical Journal Editors (ICMJE) criteria.

The study results will be reported in scientific and professional journals as well as presented at conferences. In the final trial report, the contributions of each author will be confirmed and recognised. Ghost authorship will not be used. A professional expert in English language editing was consulted before the submission of the study protocol and will be further consulted before the submission of the final trial report.

## Supplementary Information

Supplementary Material 1.

Supplementary Material 2.

## Data Availability

The data will be collected for the purpose of this study only. As approved by the University of Helsinki Research Ethics Committee in the Humanities and Social and Behavioural Sciences (reference code 1/2025), individual patient data will not be shared with other researchers. Metadata of the study (a general description) will be openly available in the OSF data archive (osf.io).
