# Supplementary material for: Effectiveness of an online training course to improve evidence-based leadership practices among unit leaders: study protocol for a cluster randomised two-arm controlled trial (EVILEAD)
Source: Trials. 2025 Nov 14;26:508. doi: 10.1186/s13063-025-09207-9 (PMC12619212; doi:10.1186/s13063-025-09207-9)
Supplement: Supplementary file 2 — Supplementary Material 2. [file 13063_2025_9207_MOESM2_ESM.docx]

**Additional file 2.**

Evidence-based leadership training description in the experimental group on the GREET checklist (1), based upon the TIDieR guidance (2).

| **BRIEF NAME** |
| --- |
| **1. INTERVENTION**: Evidence-based leadership online training course (experimental group). |
| **WHY** |
| **2. THEORY**: The evidence-based leadership online training course was designed based on Kirkpatrick’s (1994) model (3). The course assumes that to lead teams toward their goals and to solve leadership or clinical problems, leaders need the best available evidence combined with good self-awareness, transparency, and highly internationalised morals (4). |
| **3. LEARNING OBJECTIVES**: The online training course aims to improve the evidence-based leadership competences to make changes or solve unit problems together with staff members. |
| **4. EBP CONTENT**: The structure of the course will follow the steps of the evidence-based approach: 1) each participant will identify their specific unit problem to be solved together with the team and the unit leader will work with this problem during the course; 2) organisational data will be collated and analysed to understand the key problem of the unit; 3) scientific literature will be searched for, and critically appraised; 4) the views of stakeholders (patients, family members, etc.) will be considered along with implications; and 5) all sources of information will be collated together by the unit leader together with the team. The solution will be implemented into practice, and the actual situation will be evaluated (5).  The course includes seven modules: 1) orientation and leadership problem identification; 2) leadership problems and competencies; 3) collecting and analysing organisational information; 4) evidence from the scientific literature to support problem-solving; 5) considering stakeholders’ views; 6) implementation of evidence-based change; 7) evaluation of evidence-based change. |
| **WHAT** |
| **5. MATERIALS**: The course will be hosted on the Moodle learning platform. Each module includes specific learning material and tasks (slideshow presentations, scientific articles). The course material will only be accessible to the study participants assigned to the intervention group. They will be able to access the course using computers at work or home. |
| **6. EDUCATIONAL STRATEGIES**: Cognitive gains related to specific topics are supported by reading material (slideshow presentations, scientific articles). Peer-group discussions will be used to increase self-awareness and opportunities for peer-supporting, planning and implementing evidence-based solutions into daily practice. In addition, transparency and ethical sensitivity will be encouraged through small-group discussions (4). The impact of changes based on the participants’ course work will be assessed. Short assignments (max 500 words) will help demonstrate learning. All course tasks will be arranged so that unit leaders will work together their team. Self-reflection will ensure a reflective learning approach; this will build confidence among the nurse leaders to use evidence in their work, while also supporting their self-efficacy and self-esteem. Tutors will monitor the participants’ progress with a structured feedback template. This will provide uniform evaluations of the submitted assignments.  During the learning process, each participant will identify a specific leadership problem to work on during the course; this will act as a reaction for change. Second, learning will materialise through improving knowledge levels; each participant will familiarise themselves with the learning material on the learning platform. Participants will also be supported in group discussions and with encouraging feedback offered by tutors with the aim of keeping the participants’ attitudes toward evidence-based approach positive. Third, the participants will gain hands-on skills in how to seek out evidence-based scientific literature. They will also combine information based on organisational information and stakeholder analysis to gain a deeper understanding of the problem to be solved. Fourth, participants will implement their new design solution for their problem into practice and evaluate its impact. |
| **7. INCENTIVES**: Participation in the online training course may require time and effort from supervisors outside working hours. The practical exercises included in the course are designed to align with the daily activities of the workplace. No incentives will be given for participants. |
| **WHO** |
| **8. INSTRUCTORS**: The person responsible for the research and the members of the research team have extensive experience in implementing online courses designed for professionals. Trained tutors with an academic and/or healthcare professional background will be responsible for mentoring each module. The course will be run in small groups with unit leaders (15 participants in each group). Tutors (1/group) will follow the participants’ (and their team’s) progress, answer any practical questions related to the course via group chat, give feedback on the participants’ (unit leaders) assignments, monitor participants’ and staff’s activity, and support written peer discussions. The tutors will be supervised by the principal investigator. |
| **HOW** |
| **9. DELIVERY**: Evidence-based leadership training will be delivered online in groups (max 15 participants). Participants will be able to study at work or at home according to a pre-structured schedule. |
| **WHERE** |
| **10. ENVIRONMENT**: The online training course will be delivered at work and/or in free time. |
| **WHEN and HOW MUCH TIME** |
| **11. SCHEDULE**: The active study time of the course includes seven modules and a final assessment task to be completed in seven months. The duration of each module varies from one to six weeks depending on the learning activity. |
| **12.** Participants will review the course material and complete assignments at their own pace, ensuring that each module is finished within the designated timeframe.  Tutors will provide individual written feedback on participants’ assignments in each module, using a structured template. Given that each module may have up to 15 assignments, tutors are expected to spend approximately 10–15 minutes per assignment reviewing and writing feedback. |
| **PLANNED CHANGES** |
| **13.** N/A |
| **UNPLANNED CHANGES** |
| **14.** N/A |
| **HOW WELL** |
| **15. ATTENDANCE**: Feasibility and fidelity of the intervention will be assessed by calculating the number of times participants log in to the learning platform, the overall number of completed course tasks, the number of tasks completed by each participant, and the drop-out rate by the number of participants who leave the study early. The tutors will closely monitor the submission of assignments to ensure that tasks are completed according to the established schedule. The tutors will send three individual reminders during each module to encourage participants to join and finalise their assignments. |
| **16.** The delivery of materials and educational strategies will be tracked by ensuring that each module is made available on the Moodle platform according to the predetermined schedule. The materials for each module, including readings, PowerPoint presentations with recordings, and other resources, will be unlocked and accessible to the participants at the start of the assigned week for each module as planned in the curriculum. |
| **17**. N/A |

**References**

1. Phillips AC, Lewis LK, McEvoy MP, Galipeau J, Glasziou P, Moher D, Tilson JK, Williams MT. Development and validation of the guideline for reporting evidence-based practice educational interventions and teaching (GREET). BMC Med Educ. 2016 Sep 6;16(1):237. doi:10.1186/s12909-016-0759-1.

2. Hoffmann TC, Glasziou PP, Boutron I, Milne R, Perera R, Moher D, Altman DG, Barbour V, Macdonald H, Johnston M, Lamb SE, Dixon-Woods M, McCulloch P, Wyatt JC, Chan AW, Michie S. Better reporting of interventions: template for intervention description and replication (TIDieR) checklist and guide. BMJ. 2014 Mar 7;348:g1687. doi:10.1136/bmj.g1687.

3. Kirkpatrick DL. Evaluating training programs: the four levels. San Francisco: Berrett-Koehler Publishers; 1994.

4. Kernis MH, Goldman BM. A multicomponent conceptualization of authenticity: theory and research. Adv Exp Soc Psychol. 2006;38:283–357. doi:10.1016/S0065-2601(06)38006-9.

5. Barends E, Rousseau DM, Briner RB. Evidence-based management, The basic principles. Amsterdam: Center for Evidence-Based Management; 2014 [cited 2024 Sep 13]. Available from: <https://cebma.org/assets/Uploads/Evidence-Based-Practice-The-Basic-Principles.pdf>
